# Reevaluation of the role of parallel fiber synapses in delay eyeblink conditioning in mice using Cbln1 as a tool

**DOI:** 10.3389/fncir.2013.00180

**Published:** 2013-11-15

**Authors:** Kyoichi Emi, Wataru Kakegawa, Eriko Miura, Aya Ito-Ishida, Kazuhisa Kohda, Michisuke Yuzaki

**Affiliations:** ^1^Department of Physiology, School of Medicine, Keio UniversityShinjuku-ku, Tokyo, Japan; ^2^Core Research for Evolutional Science and Technology, Japan Science and Technology CorporationKawaguchi, Saitama, Japan

**Keywords:** mouse, motor learning, cerebellum, long-term depression, Purkinje cell

## Abstract

The delay eyeblink conditioning (EBC) is a cerebellum-dependent type of associative motor learning. However, the exact roles played by the various cerebellar synapses, as well as the underlying molecular mechanisms, remain to be determined. It is also unclear whether long-term potentiation (LTP) or long-term depression (LTD) at parallel fiber (PF)–Purkinje cell (PC) synapses is involved in EBC. In this study, to clarify the role of PF synapses in the delay EBC, we used mice in which a gene encoding Cbln1 was disrupted (*cbln1*^-/-^ mice), which display severe reduction of PF–PC synapses. We showed that delay EBC was impaired in *cbln1*^-/-^ mice. Although PF-LTD was impaired, PF-LTP was normally induced in *cbln1*^-/-^ mice. A single recombinant Cbln1 injection to the cerebellar cortex *in vivo* completely, though transiently, restored the morphology and function of PF–PC synapses and delay EBC in *cbln1*^-/-^ mice. Interestingly, the *cbln1*^-/-^ mice retained the memory for at least 30 days, after the Cbln1 injection’s effect on PF synapses had abated. Furthermore, delay EBC memory could be extinguished even after the Cbln1 injection’s effect were lost. These results indicate that intact PF–PC synapses and PF-LTD, not PF-LTP, are necessary to acquire delay EBC in mice. In contrast, extracerebellar structures or remaining PF–PC synapses in *cbln1*^-/-^ mice may be sufficient for the expression, maintenance, and extinction of its memory trace.

## INTRODUCTION

**FIGURE 1:**
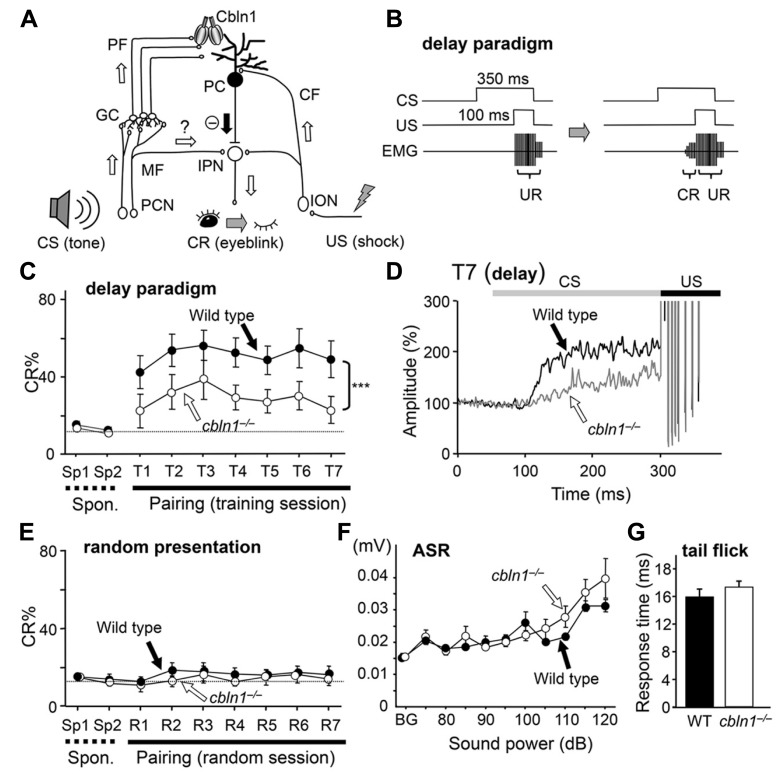
**Delay eyeblink conditioning (EBC) is impaired in *cbln1*^-/-^ mice.** (**A**) A diagram showing the cerebellar circuits responsible for delay EBC. A conditioned stimulus (CS) tone increases the activities of precerebellar nuclei (PCN), which send MF to granule cells (GCs). Some MFs may also innervate the interpositus nucleus (IPN). Parallel fibers (PFs), which are GC axons, secrete Cbln1, which regulates the integrity and plasticity of PF–Purkinje cell (PC) synapses. An unconditioned stimulus (US) is mediated by the activities of the inferior olivary nuclei (ION), which send climbing fibers (CFs) to the IPN and PCs. **(B)** A diagram showing the delay EBC paradigm. A CS tone (350 ms) precedes and co-terminates with a US (100 ms). Mice were trained for one session per day for 7 days. Associative learning was established when conditioned response (CR), detected by electromyogram (EMG), was observed before the unconditioned eyeblink response (UR). **(C)** Impaired delay EBC in *cbln1*^-/-^ mice. The CR% was significantly smaller for *cbln1*^-/-^ than wild-type mice (*p* < 0.01, two-way repeated measures ANOVA, *n* = 8 mice for each group). T1–T7, training sessions; Sp1 and Sp2, spontaneous eyeblink responses before training. The *Y*-axis gives the percentage of trials showing positive CR. **(D)** Averaged EMG amplitude on the last day of training (T7). The EMG amplitudes were normalized to those during the initial 30 ms of CS. *n* = 8 for each group. **(E)** CRs by pseudo-random test. CS and US stimuli were randomly presented to wild-type and *cbln1*^-/-^ mice. There was no significant difference in CR% between two groups (*p* > 0.1). **(F)** Acoustic startle response (ASR) test. There was no significant difference in the amplitude between wild-type and *cbln1*^-/-^ mice (*p* > 0.5, by two-way repeated measures ANOVA, *n* = 8 for each group). **(G)** A tail-flick test. No significant difference was found in response time to nociceptive stimuli between wild-type and *cbln1*^-/-^ mice (*p* > 0.5, by Mann–Whitney’s *U* test, *n* = 8 for each group). The dotted lines in C and E correspond to the percentage of CR-like activities on Sp2 to indicate the baseline for learned responses.

The cerebellum is one of brain regions in which learning at the behavioral level could be directly associated with changes in neural networks at synaptic levels. The delay eyeblink conditioning (EBC) is a cerebellum-dependent type of associative motor learning extensively studied in rabbits as well as cats, ferrets, and rats (**Figure 1A**; Hesslow and Yeo, 2002; Christian and Thompson, 2003). An unconditioned stimulus (US), e.g., an air puff or weak periorbital electric shock, is applied just before the end of a conditioned stimulus (CS), such as a tone or light (**Figure 1B**). After repeated exposure to paired CS–US presentations, the animal begins to blink its eyes before the US arrives: this is the conditioned response (CR). The CS is thought to be conveyed to the cerebellum via the pontine nuclei and their mossy fiber axons, which innervate granule cells, leading to activation of Purkinje cells (PCs) via parallel fibers (PF; axons of granule cells). Some mossy fibers (MF) may also innervate deep cerebellar nuclei (DCN; Kleim et al., 2002). The US is conveyed as increased activities of climbing fibers (CFs), which originate from the inferior olive of the medulla and innervate PCs. According to the Marr–Albus–Ito theory, the neural activities of CFs convey teacher signals that induce long-term depression (LTD) of PF–PC synaptic transmission (PF-LTD), which serves as a memory trace during acquisition of EBC. In addition, mossy fiber–DCN synapses may also be involved in EBC, depending on the time course or particular aspects of memory (van Alphen and De Zeeuw, 2002; Blazquez et al., 2004; Boyden et al., 2004; Shutoh et al., 2006). The exact roles played by the various cerebellar synapses, as well as the underlying molecular mechanisms, remain to be determined at different learning phases (e.g., acquisition, expression, extinction, and saving) of delay EBC.

Recent advent of genetically modified mice has opened up the possibility to further clarify molecular mechanisms responsible for EBC. Most mutant mice, in which molecules required for PF-LTD induction are modified, display impaired EBC, supporting the notion that PF-LTD is responsible for motor learning *in vivo* (Yuzaki, 2012). Recently, however, three lines of genetically modified mice (Schonewille et al., 2011), in which PF-LTD was abolished, were reported to display normal EBC (Schonewille et al., 2011). Conversely, PC–specific calcineurin knockout mice, which had a normal LTD but impaired long-term potentiation (LTP at PF–PC synapses, showed impaired EBC (Schonewille et al., 2010). Thus, instead of PF-LTD, PF-LTP is proposed as an alternative substrate for motor learning (Porrill and Dean, 2008; Schonewille et al., 2010). However, in most genetically engineered mice where molecules required for PF-LTD induction were modified, PF-LTP has not yet been systemically investigated (Yuzaki, 2012). In addition, compensatory pathways could easily kick in when specific genes are knocked out or expressed throughout life. For example, while PF-LTD is normal in cerebellar slices prepared from PC–specific calcineurin knockout mice (Schonewille et al., 2010), inclusion of calcineurin inhibitory peptides in the patch pipette completely blocks LTD induction in wild-type (WT) cerebellar slices (Fujiwara et al., 2007; Nomura et al., 2012). Furthermore, the same gene product is often shared by different synapses in the targeted neuron. For example, since protein kinase C (PKC) is ubiquitous in PCs, PF–, CF–, and interneuron–PC synapses may all be affected in L7-PKCI transgenic mice (De Zeeuw et al., 1998). Therefore, temporally specific ablation or expression of gene products at specific synapses will be required to clarify the role of PF-LTD in motor learning.

Cerebellin precursor protein 1 (Cbln1) is a C1q family protein, which is produced and secreted from cerebellar granule cells (Hirai et al., 2005). Cbln1 binds to its postsynaptic receptor, glutamate receptor delta 2 (GluD2), expressed in PCs, and its presynaptic receptor, neurexin (Matsuda et al., 2010; Uemura et al., 2010; Matsuda and Yuzaki, 2011). Mice in which a gene encoding Cbln1 is disrupted (*cbln1*^-/-^ mice) exhibit ~ 80% reduction of PF–PC synapses (Hirai et al., 2005). Abnormalities are also found at CF–PC synapses; redundant CFs are gradually eliminated until a one-to-one relation-ship is established by the end of the third postnatal week (PW) In WT PCs, whereas supernumerary CFs remain to innervate *cbln1*^-/-^ PCs even in adulthood (Hirai et al., 2005). A single injection of recombinant Cbln1 into the subarachnoid space over the cerebellum rapidly (in ~ 48 h) restores PF synapses in adult *cbln1*^-/-^ mice without affecting CF synapses (Ito-Ishida et al., 2008). Nevertheless, ataxic phenotype of *cbln1*^-/-^ mice is completely but transiently rescued, indicating that Cbln1 is necessary and sufficient for the formation of PF–PC synapses and gross motor performance *in vivo*. Interestingly, PF-LTD is also impaired in the *cbln1*^-/-^ cerebellum, indicating that Cbln1 is not only required for formation and maintenance of PF synapses, but also for regulation of synaptic plasticity in the remaining PF synapses (Hirai et al., 2005). However, it has been untested whether delay EBC and PF-LTP could be normally induced in *cbln1*^-/-^ mice, and whether Cbln1 injection could modulate these phenomena *in vivo*.

In this study, to clarify the role of PF synapses at different learning phases, we examined the delay EBC in *cbln1*^-/-^ mice after injection of recombinant Cbln1 at different time points. We showed that delay EBC, but not trace EBC (Kishimoto et al., 2001b; Brown et al., 2010), was impaired in *cbln1*^-/-^ mice. Although PF-LTD was impaired, PF-LTP was normally induced in *cbln1*^-/-^ mice. A single recombinant Cbln1 injection completely, though transiently, restored PF-LTD and delay EBC in *cbln1*^-/-^ mice. Interestingly, the *cbln1*^-/-^ mice retained the CR for at least 30 d, after the Cbln1 injection’s effect on PF synapses and PF-LTD induction had abated. Furthermore, delay EBC extinction by exposure to the unpaired CS succeeded, even after the Cbln1 injection’s effect were lost. These results indicate that intact PF–PC synapses and PF-LTD, not PF-LTP, are necessary to acquire delay EBC in mice. In contrast, extracerebellar structures or remaining PF–PC synapses in *cbln1*^-/-^ mice may be sufficient for the expression, maintenance, and extinction of its memory trace.

## MATERIALS AND METHODS

### MICE

This study used *cbln1*^-/-^ mice on a C57BL6/J genetic background and their WT littermates. The mice were housed individually on a 12-h light/dark cycle, and given ad-lib access to food and water. All experiments were performed in accordance with the guidelines established by the Institutional Animal Investigation Committee at the Keio University School of Medicine. All efforts were made to minimize the use of animals and to optimize their comfort.

### PREPARATION OF RECOMBINANT HA-CBLN1

Recombinant hemagglutinin-conjugated Cbln1 (HA-Cbln1) was prepared as described previously (Ito-Ishida et al., 2008). Briefly, pCAGGS-HA-Cbln1 was transfected into human embryonic kidney 293 (HEK293) cells using a calcium phosphate method (CellPhect, GE Healthcare Inc., USA). After 12 h, the culture medium was replaced with chemically defined 293 medium (Invitrogen Inc., USA). Two days later, the medium was collected and concentrated using centrifugal filtering devices (Centriplus YM-10 and Microcon YM-100, Millipore Inc., USA). The concentrated medium was washed twice with 500 μl phosphate-buffered saline (PBS) and the final volume of the medium was adjusted to 50 μl. Protein was measured using a BCA protein assay kit (Thermo Fischer Scientific, USA). The control solution was prepared from the medium of non-transfected HEK293 cells.

### HA-CBLN1 INJECTION

Male *cbln1*^-/-^ mice at PW 7–10 were anesthetized with an intraperitoneal injection of ketamine [80 mg/kg body weight (BW)] and xylazine 20 mg/kg BW (Sigma-Aldrich Inc., USA). A subarachnoid injection of HA-Cbln1 was given as described previously (Ito-Ishida et al., 2008; Matsuda et al., 2010). In brief, a small hole in the occipital bone was made with a dental drill, and the dura mater was ablated. A 33-gage microsyringe needle was inserted onto the surface of the simplex lobules (hemispheric lobule VI, HVI) of the cerebellum. HA-Cbln1 (1 μg/g BW) was injected into the subarachnoid space bilaterally, at a rate of 40 μl/h for 15 min. To prevent the infused solution from leaking, the injection site was sealed with a silicone elastomer (Kwik-Sil; World Precision Instruments, USA).

### SURGICAL PROCEDURES FOR EYEBLINK CONDITIONING

Four Teflon-coated stainless steel wires (A-M Systems, Inc. Carlsborg, WA, USA) were implanted into the left eyelid of the mouse under deep anesthesia. Two wires were used to record electromyograms (EMG), and the remaining two to deliver weak electric shocks. The wires were soldered to connector pins, which were secured to small stainless steel screws fixed on the skull with dental acrylic resin. After surgery, the animals were returned to their home cages to recover for 2 days.

### EYEBLINK CONDITIONING

Mice were subjected to EBC training as described previously (Kishimoto et al., 2001a; Kakegawa et al., 2008). Each mouse was put in a cylindrical Plexiglas container (10 cm in diameter and 25 cm in height) in a sound- and light-attenuating chamber. The connector pins fixed to the mouse’s skull were connected to a flexible cable, which allowed the mouse to move freely during the conditioning trials. EMG activity was recorded through a band-pass filtered amplifier (0.15 and 1.0 kHz, MEG-5200, Nihonkoden, Japan) and fed into a computer at a sampling rate of 10 kHz. A 100-Hz periorbital electrical shock (100 ms) and a 1-kHz tone (85 dB, 350 ms) were used as unconditioned (US) and conditioned stimuli (CS), respectively. We employed this relatively high intensity CS condition (Boele et al., 2010) since WT mice showed stable learning throughout the training sessions (Sakamoto and Endo, 2010). Spontaneous eyeblink activities were recorded without applying US or CS on days 1 and 2.

In the delay paradigm, the CS tone preceded and co-terminated with the US shock. Each training session consisted of 90 CS–US paired trials and 10 CS-only trials that were presented every tenth trial. There was one training session per day, on days 3–9. The trials were separated by randomized inter-trial intervals from 20 to 40 s duration. The US intensity was adjusted daily to elicit an eyeblink/head turn response to obtain a constant amplitude of unconditioned response (UR). In the trace paradigm study, the US started 0, 250, or 500 ms after the CS ended. The trace conditioning experiment also involved 7 days of training sessions (Kishimoto et al., 2001b).

Electromyogram signals were analyzed offline by custom software (Microsoft Visual Basic 2003; Kakegawa et al., 2008). The EMG wave analysis algorithm has been described previously (Kishimoto et al., 2001a). Briefly, the maximum amplitude of EMG signals in each 1-ms bin represented the EMG amplitude during that time window. We calculated the average and the standard deviation (SD) of EMG amplitude during the 300 ms before CS onset in 100 trials. The averaged EMG amplitude plus 1 SD was defined as a “threshold value.” In each trial, the “baseline value” was calculated by averaging EMG amplitude that exceeded the threshold value during the 300 ms prior to the CS onset. In addition, we calculated the “startle value” by averaging EMG amplitude that exceeded the threshold value during the 30 ms after the CS. If the baseline and the startle values were less than 10% and 30% of the threshold value, respectively, the trial was considered valid. In the valid trial, the CR value was calculated by averaging EMG amplitude 200 ms before the US. In CS-only trials, the CR values were obtained by averaging the EMG amplitude over the period from 200 ms before to 100 ms after the expected US onset. If the CR value exceeded 1% of the threshold and was more than twice the baseline value, we regarded the trial as CR positive. The CR% was calculated for each mouse as the percentage of CR-positive trials among the valid trials in each training session. The CR% values for the individual mice were averaged to obtain the group CR% for each training session. In addition, before training session was started, the CR% values were similarly calculated in 100 trials without applying CS or US to estimate CR-like activities in each mouse.

To test retention and extinction of the delay EBC, we first chose mice showing CR% greater than 60%. Each mouse was left undisturbed for 30 days in its home cage after acquisition training and then tested for retention of the conditioned memory. For the retention test, the mice were given a 1-day trial session consisting of 90 CS–US paired trials and 10 CS-only trials (presented every 10th trial) for 1 day. For the extinction test, mice were given 100 CS-only trials for 4 days (Kishimoto et al., 2001b; Thompson and Steinmetz, 2009). The conditioning experiments were all carried out during the light phase of the light/dark cycle. The EBC training, memory testing, and extinction were performed by an experimenter blinded to each mouse’s genotype.

### ACOUSTIC STARTLE REFLEX TEST

The acoustic startle reflex test was performed as described previously (Emi et al., 2011). Each mouse was placed in the acoustic startle device (SRLAB, San Diego Instruments, USA) consisted of a Plexiglas cylinder (5-cm in outer diameter) in a sound-attenuated box (approximately 20-cm^2^) with a high frequency loudspeaker (28 cm above the cylinder) that produced both a continuous background noise (70 dB) and the acoustic stimuli (bursts of white noise between 4 and 14 kHz). Movements of the mouse in the cylinder were transduced by a piezoelectric accelerometer attached to the bottom of the platform, and digitized. The maximal peak-to-peak amplitude from the piezoelectric sensor was used to determine the magnitude of the acoustic startle response (ASR). The session was initiated with a 5-min acclimation period followed by four different sound stimuli (75, 80, 85, 90 dB) presented in a random order with random intertrial intervals (10–20 s; 15 s on average). All trials were repeated 10 times.

### TALE FLICK TEST

Standard nocifensive reflex test (tail flick test) was performed on mice using a radiant heat apparatus (IITC, Woodland Hills, CA, USA). Mice were restrained in a Plexiglas tube and allowed to acclimate for 5–10 min before testing. Ten successive trials (60 s apart) with each mouse were averaged to obtain a mean value for each mouse.

### IMMUNOBLOTTING AND IMMUNOHISTOCHEMISTRY

Two days after the HA-Cbln1 injection, the whole cerebellum of a *cbln1*^-/-^ mouse was homogenized in a buffer containing 10 mM Tris-HCl, 1 mM EDTA, and 0.32 M sucrose (pH 7.5), and was centrifuged at 800 × *g* for 10 min at 4°C. The supernatant was centrifuged at 12,000 × *g* for 20 min at 4°C, and the pellet (P2 fraction) was solubilized in 2× SDS sample buffer. The protein concentration was measured using a BCA protein assay kit. Equal protein amounts were subjected to SDS–polyacrylamide gel electrophoresis (PAGE) and blotted onto a PVDF membrane (Hybond-P, GE Healthcare, USA). HA-Cbln1 was detected with an enhanced chemiluminescence system (Immobilon Western, Millipore Inc., USA) and quantified using an LAS-3000 CCD Imaging System (Fujifilm Co Ltd., Japan).

For immunohistochemical analysis, mice were fixed under deep anesthesia by cardiac perfusion with 0.1 M sodium phosphate buffer (PB), pH 7.4, containing 4% paraformaldehyde (4% PFA/PB); the cerebellum was then removed and soaked in 4% PFA/PB for 4 h. After rinsing the specimens with PBS, parasagittal slices (100 μm) were prepared using a microslicer (DTK-2000; Dosaka, Japan) and were permeabilized with 0.2% Triton X-100 in PBS with 2% normal goat serum and 2% bovine serum albumin for 6 h at 4°C. Immunohistochemical staining was performed using anti-HA antibody (1:1000; Covance Research Products), followed by incubation with Alexa546-conjugated secondary antibodies (1:1000; Invitrogen) and fluorescent Nissl stains (Neurotrace; 1:300, Invitrogen). The stained slices were viewed using a confocal laser-scanning microscope (Fluoview; Olympus).

### ELECTROPHYSIOLOGY

Parasagittal cerebellar slices (200 μm thick) were prepared from adult *cbln1*^-/-^ mice at 2 or 30 days after the recombinant HA-Cbln1 injection, as described previously (Kakegawa et al., 2008). Briefly, whole-cell patch-clamp recordings were made from visually identified PC s using a 60× water-immersion objective attached to an upright microscope (BX51WI; Olympus Optical, Tokyo, Japan) at room temperature. The patch pipette resistance was 3–5 Mømega when filled with the following intracellular solution (in millimoles): 65 Cs-methanesulfonate, 65 K-gluconate, 20 HEPES, 10 KCl, 1 MgCl_2_, 4 Na_2_ATP, 1 Na_2_GTP, 5 sucrose, and 0.4 EGTA, pH 7.25 (295 mOsm/kg) for LTD experiment and 130 K-gluconate, 10 KCl, 10 HEPES 1 MgCl_2_, 4 Na_2_ATP, 1 Na_2_GTP and 16 sucrose, pH 7.25 (295 mOsm/kg) for LTP experiment. The solution used for slice storage and recording contained (in millimoles) 125 NaCl, 2.5 KCl, 2 CaCl_2_, 1 MgCl_2_, 1.25 NaH_2_PO_4_, 26 NaHCO_3_, and 10 D-glucose, bubbled continuously with a mixture of 95% O_2_ and 5% CO_2_. To block inhibitory synaptic transmission, 100 μM picrotoxin (P1675, Sigma-Aldrich Inc., MI, USA) was always added to the saline.

To induce synaptic plasticity at PF–PC synapses, PF–evoked excitatory postsynaptic currents (PF–EPSCs) were first recorded successively at a frequency of 0.1 Hz from PCs clamped at –80 mV. After stable PF–EPSCs were observed for at least 10 min, a conjunctive stimulation (CJ-stim) composed of 30 single PF stimuli together with 200-ms depolarizing pulses from a holding potential of –60 to +20 mV was applied to induce LTD (Kakegawa et al., 2008). For induction of postsynaptic LTP, 300 times of PF stimuli were applied at a frequency of 1 Hz in the current-clamp mode (Lev-Ram et al., 2002). Access resistances were monitored every 10 s by measuring the peak currents in response to 2-mV, 50-ms hyperpolarizing steps throughout the experiments; the measurements were discarded if the resistance changed by more than 20% of its original value. EPSCs were recorded using an Axopatch 200B amplifier (Molecular Devices Inc., USA), and pClamp software (version 9.2, Molecular Devices Inc., USA) was used for data acquisition and analysis. The signals were filtered at 1 kHz and digitized at 4 kHz.

### ELECTRON MICROSCOPIC STUDIES

Samples for electron microscopic studies were prepared as previously described (Ito-Ishida et al., 2008). Mice under deep pentobarbital anesthesia were perfused transcardially with 2% paraformaldehyde/2% glutaraldehyde in 0.1 M PB (pH 7.2). Parasagittal sections (300 μm thick) of the simplex lobule of the cerebellum were postfixed and stained for 2 h with 1% OsO_4_ in 0.1 M PB. The sections were block-stained in 1% aqueous uranyl acetate solution, dehydrated with graded alcohols, and embedded in Epon 812. Ultrathin sections (70 nm thick) were made with an ultramicrotome (Leica Microsystems Co Ltd., Germany) and stained with 2% uranyl acetate for 5 min, followed by mixed lead solution for 2 min. Electron micrographs of the molecular layer were taken (H7100, Hitachi Co Ltd., Japan) at 4,000× and printed at 16,000×. The numbers of normal and free PC spines (which contained postsynaptic density-like condensations but lacked presynaptic contact) were counted using 10 micrographs per mouse, taken randomly (Hirai et al., 2005).

#### Statistical analysis

All data are presented as mean ± SEM. Statistical significance was determined by Mann–Whitney’s *U* test, the Fisher’s exact test, or an ANOVA, followed by the Bonferroni’s test for multiple comparisons, using the SPSS program (Ver.15, SPSS Co Ltd., Japan). The difference was considered as significant when the *p*-value was less than 0.05.

## RESULTS

### DELAY, BUT NOT TRACE, EYEBLINK CONDITIONING WAS IMPAIRED IN *CBLN1*^-/-^ MICE

To examine motor learning in *cbln1*^-/-^ mice, we employed an EBC delay paradigm, in which a CS tone preceded and co-terminated with a US shock (**Figures 1A, B**). Although both *cbln1*^-/-^ and WT mice showed increased occurrence of conditioned responses (CRs) after several training sessions, there were significant differences in acquisition of CRs between *cbln1*^-/-^ and WT mice during the 7-day training period (*p* < 0.01 for genotype, a two-way repeated-measures ANOVA; **Figure 1C**). Similarly, in the delay EBC paradigm, the average CR% saturates at 20–40% in mice with various cerebellar mutations (Chen et al., 1996; Qiao et al., 1998; Kishimoto et al., 2001b; Wada et al., 2007; Emi et al., 2011), although it can reach 50–70% in WT mice. As reported previously for EBC in mice (Boele et al., 2010), eyeblink responses consist of short-latency responses (SLRs), which are likely mediated by amygdala, and a later responses mediated by the cerebellum (Sakamoto and Endo, 2010). Indeed, the averaged EMG amplitudes before the US in the seventh training sessions show SLRs in both *cbln1*^-/-^ and WT mice (**Figure 1D**), indicating that the remaining learning may be mediated by extracerebellar structures. Nevertheless, the averaged EMG amplitudes before the US were significantly lower in *cbln1*^-/-^ mice than in WT mice, suggesting that Cbln1, which is mostly expressed in cerebellar granule cells but not in amygdala (Miura et al., 2006), may be involved in the delay EBC.

To rule out the possibility that mice acquired conditioned responses by nonassociative learning, we next performed random presentation protocol. When the CS and US were randomly presented, the WT nor the *cbln1*^-/-^ mice acquired conditioned responses (*p* > 0.1, a two-way repeated measures ANOVA; **Figure 1E**), excluding the possibility of non-associative learning. There were no differences in ASRs (*p* > 0.5, a two-way repeated measures ANOVA, **Figure 1F**) or tail-flick responses to heat (*p* > 0.1, Mann–Whitney’s *U* test; **Figure 1G**) between the two genotypes. The US intensities required to elicit an eyeblink during conditioning did not differ between two genotypes (data not shown), indicating that the *cbln1*^-/-^ mice had no gross sensory system defects. Thus, the delay EBC paradigm, a form of associative motor learning, was likely impaired in the *cbln1*^-/-^ mice.

**FIGURE 2:**
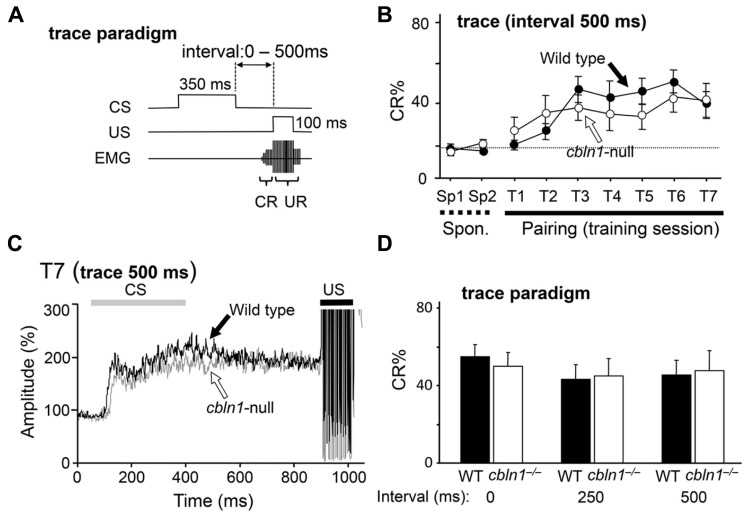
**Trace eyeblink conditioning (EBC) is intact in *cbln1*^-/-^ mice.** **(A)** Diagram showing trace EBC. An unconditioned stimulus (US, 100 ms) was applied 500 ms after the end of the conditioned stimulus (CS, 350 ms) tone. Mice were trained for 7 days, one session per day. Associative learning was established when conditioned responses (CR), detected by electromyogram (EMG) were observed before unconditioned eyeblink responses (UR). **(B)** Intact trace EBC in *cbln1*^-/-^ mice. No significant difference in CR% was observed between wild-type and *cbln1*^-/-^ mice (*p* > 0.1, a two-way repeated measures ANOVA, *n* = 10 mice for each group). T1–T7, training sessions; Sp1 and Sp2, spontaneous eyeblink responses before training. The *Y*-axis indicates the percentage of trials showing CR. The dotted line corresponds to the percentage of CR-like activities on Sp2 to indicate the baselin e for learned responses. **(C)** Averaged EMG amplitude on the last day of training (T7). The EMG amplitudes were normalized to those during the initial 30 ms of CS; *n* = 10 for each group. No significant differences were observed between the two groups. **(D)** The effect of different time intervals on trace EBC. The interval between the end of the CS and the beginning of the US was shortened from 500 ms to 250 or 0 ms. The CR% on the last day of training (T7) is shown for each genotype. No significant CR% difference was observed for 500, 250, and 0 ms intervals between wild-type and *cbln1*^-/-^ mice (*p* > 0.1, two-way repeated measures ANOVA followed by Bonferroni’s correction, *n* = 8 for each genotype).

The trace EBC paradigm, in which a stimulus-free interval intervenes between the CS and the US (**Figure 2A**), requires extracerebellar structures and less depends on the cerebellar cortex in mice (Kishimoto et al., 2001b; Brown et al., 2010). Thus, we examined whether *cbln1*^-/-^ mice could acquire associative memory by the EBC trace paradigm. First, the animals were given a CS, followed by a US 500 ms after the end of the CS, 100 times per day, for 7 days. In both WT and *cbln1*^-/-^ mice, the CR% gradually increased, with no significant differences between the genotypes (*p* > 0.1, a two-way repeated measures ANOVA; **Figure 2B**). Similarly, the averaged EMG amplitudes between the CS and US in the seventh training session were similar between the two genotypes (**Figure 2C**). We also used shorter interstimulus intervals between the CS and US and found that WT and *cbln1*^-/-^ mice showed a similar CR% at the end of the seventh training session (*p* > 0.1 for both 0- and 250-ms intervals, two-way repeated measures ANOVA followed by Bonferroni’s correction; **Figure 2D**). Thus, that *cbln1*^-/-^ mice are able to acquire conditioned responses in EBC depending on the training protocol. These results are consistent with an earlier report that mice lacking GluD2, a receptor for Cbln1, showed normal trace EBC (Kishimoto et al., 2001a, b). Together, the delay EBC paradigm was specifically impaired in *cbln1*^-/-^ mice.

### INJECTED CBLN1 TRANSIENTLY RESTORED EYEBLINK CONDITIONING IN *CBLN1*^-/-^ MICE

**FIGURE 3:**
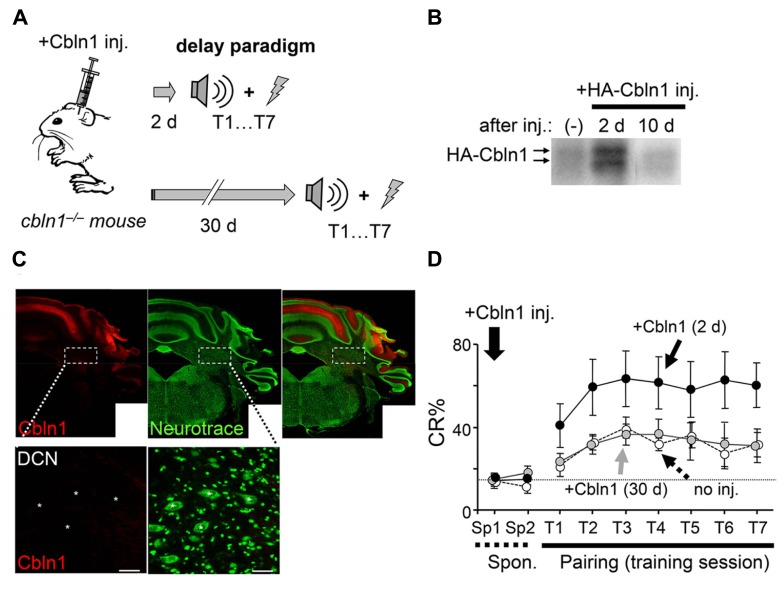
**Delay eyeblink conditioning (EBC) in adult *cbln1*^-/-^ mice improves after a cerebellar subarachnoid Cbln1 injection.** **(A)** A diagram showing a sequence of experiments. HA-Cbln1 (1 μg/g body weight) was injected into the subarachnoid spaces over the bilateral HVI lobes of 7- to 10-week-old *cbln1*^-/-^ mice. The mice were trained using the delay EBC paradigm beginning 2 or 30 days after the injection. **(B)** HA-Cbln1 disappears from the cerebellum by 10 d after injection. Immunoblotting with an antibody against HA was used to detect injected HA-Cbln1 in the P2 fraction of the whole cerebellum before (-), 2 days, and 10 days after the injection. Arrows indicate bands corresponding to HA-Cbln1. The blot represents results from *n* = 3 mice. **(C)** Distribution of injected Cbln1. Cerebellar slices were stained with anti-HA antibody (red) to detect HA-Cbln1 24 h after the injection. Neurons visualized with fluorescent Nissl stains (Neurotrace; green). The injected Cbln1 was localized to the cerebellar cortex. Asterisks in the enlarged images indicate the deep cerebellar nucleus (DCN). Scale bars, 20 μm. **(D)** Cbln1 transiently rescued the impaired delay EBC in *cbln1*^-/-^ mice. The CR% increased significantly in *cbln1*^-/-^ mice trained 2 days after a Cbln1 injection [+Cbln1 (2d)] compared to those trained 30 days after [+Cbln1 (30 days)] the injection or those not treated with Cbln1 (no inj; *p* < 0.01, a two-way repeated measures ANOVA, *n* = 8 for each group). T1-T7, training sessions; Sp1 and Sp2, spontaneous eyeblink responses before training. The dotted line corresponds to the average percentage of CR-like activities on Sp2 to indicate the baseline for learned responses.

Although Cbln1 is mostly expressed in cerebellar granule cells, it is also expressed in the DCN and extracerebellar structures, such as the olfactory bulb, entorhinal cortex, and thalamus (Miura et al., 2006). To rule out the involvement of these structures in the delay EBC, we examined whether the delay EBC defect in the *cbln1*^-/-^ mice could be rescued by applying Cbln1 to PF–PC synapses. We previously showed that a single injection of recombinant Cbln1 into the subarachnoid space over the vermis rapidly but transiently restored PF–PC synapses and motor coordination in *cbln1*^-/-^ mice, as measured by the rotor-rod test (Ito-Ishida et al., 2008). Since delay EBC is likely mediated by the bilateral HVI (Miller et al., 2003; Plakke et al., 2007; Sun, 2012), we performed bilateral injections of Cbln1 (a total of 1 μg/g body weight) into the subarachnoid spaces over each HVI (**Figure 3A**) of 7- to 10-week-old *cbln1*^-/-^ mice. We detected recombinant Cbln1 in the membrane fraction of the whole cerebellum by immunoblot analysis at 2 days, but not 10 days, after the injection (**Figure 3B**). Immunohistochemical analysis of coronal cerebellar sections from *cbln1*^-/-^ mice taken 2 days after the Cbln1 injection found recombinant Cbln1 throughout the cerebellar cortex including HVI, but not in the DCN region (**Figure 3C**). When *cbln1*^-/-^ mice were subjected to delay EBC starting 2 days after a Cbln1 injection, they show significantly better CR% than *cbln1*^-/-^ mice that did not receive the injection (*p* < 0.01 for genotype, a two-way repeated measures ANOVA; **Figure 3D**) and comparable to that achieved by WT mice (**Figure 1C**), indicating that the local Cbln1 injection was able to rapidly restore motor learning defects in the *cbln1*^-/-^ mice.

To examine whether Cbln1’s effect on delay EBC was as transient as its effect on motor performance (Ito-Ishida et al., 2008), we next subjected *cbln1*^-/-^ mice to delay EBC 30 days after the injection, when the injected Cbln1 was no longer detectable (**Figure 3B**). There were no significant differences in CR% between *cbln1*^-/-^ mice that did or did not receive Cbln1 (*p* > 0.1, a two-way repeated measures ANOVA; **Figure 3D**), showing that the effect of the injected Cbln1 on the impaired EBC in *cbln1*^-/-^ mice was indeed transient. Together, these results indicate that the lack of Cbln1 in the cerebellum was responsible for the defective delay EBC in the *cbln1*^-/-^ mice and that this phenotype was rapidly but transiently rescued by the recombinant Cbln1 injection.

### CBLN1 RESTORED PF– BUT NOT CF–PURKINJE CELL SYNAPSES IN *CBLN1*^-/-^ MICE

**FIGURE 4:**
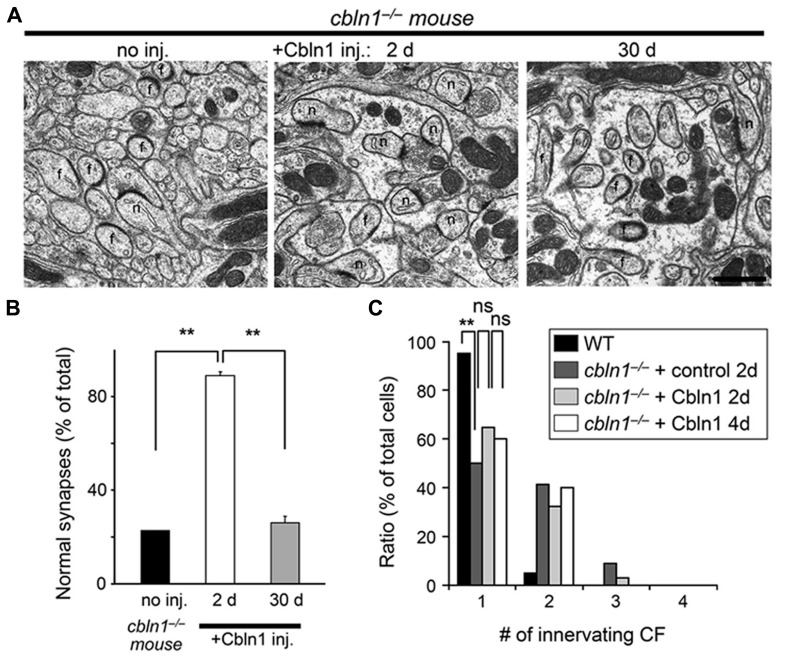
**Subarachnoidal Cbln1 injection transiently restores PF–Purkinje cell synapse structure in *cbln1*^-/-^ mice.** **(A)** Electron micrographs of the molecular layer in *cbln1*^-/-^ mice aged P42–P54 without Cbln1 injection (no inj) and at 2 or 30 days after Cbln1 injection. f, free spines without innervation by presynaptic structures; n, normal spines. Scale bar, 500 nm. **(B)** The percentage of normal synapses in *cbln1*^-/-^ mice with no injection (no inj), or 2 or 30 d after a Cbln1 injection. Significant recovery of normal synapses was observed 2 days after a Cbln1 injection, compared with no injection or their state 30 days after the injection (**p < 0.001, Mann-Whitney’s *U* test, *n* = 3 for each group). **(C)** Cbln1 injection transiently rescued the multiple CF innervation pattern in a single Purkinje cell from a *cbln1*^-/-^ mouse. The number of CF–EPSCs induced by different stimulus thresholds (0–200 μA) was counted. The percentage of Purkinje cells innervated by single CFs in wild-type (WT) mice was significantly higher than that in *cbln1*^-/-^ mice 2 days after the injection of a control solution (+control 2 days; **p < 0.00001, Fisher’s exact test). There were no significant (ns) difference between control solution and Cbln1 at 2 days after the injection (+Cbln1 2 days; *p* > 0.1, Fisher’s exact test). Similarly, there were no significant difference between 2 and 4 days after a Cbln1 injection (+Cbln1 4 days; *p* > 0.1, Fisher’s exact test).

We used electron microscopy to examine whether the bilateral Cbln1 injection also rescued morphological abnormalities observed at *cbln1*^-/-^ PF–PC synapses with a similar time-course as its rescue of delay EBC. We found that PF–PC synapse anatomical abnormalities in *cbln1*^-/-^ mice were significantly improved 2 days after bilateral Cbln1 injections (*p* < 0.001 vs. non-injected *cbln1*^-/-^ control, Mann–Whitney’s *U* test; **Figures 4A, B**): approximately 90% of the spines formed normal asymmetrical synapses with presynaptic terminals. In contrast, only 20–25% of synapses showed normal morphology in the *cbln1*^-/-^ cerebellum 30 days after the Cbln1 injection, similar to the untreated *cbln1*^-/-^ cerebellum (**Figures 4A, B**), and confirming that Cbln1 transiently rescued PF synapse morphological abnormalities.

Unlike WT PC s, *cbln1*^-/-^ PC s remain innervated by supernumerary CFs even during adulthood (Hirai et al., 2005). Since Cbln1 and its receptor GluD2 are not expressed at CF–PC synapses (Yuzaki, 2009), this defect is likely to be indirectly caused by the loss of PF–PC synapses in *cbln1*^-/-^ mice. Thus, to examine whether bilateral injection of Cbln1 restored normal CF innervation patterns in *cbln1*^-/-^ PCs, we measured the threshold to elicit CF–EPSCs in each PC, since a single CF has a single threshold for excitation. As reported previously (Hirai et al., 2005), single EPSCs were elicited in ~ 95% of WT PC s at postnatal day 46 (P46)–P52 (*n* = 60 out of 63 cells from 5 mice), whereas only ~ 50% of age-matched *cbln1*^-/-^ PC s that were injected with Cbln1-free control solutions showed a one-to-one relationship with CFs (*n* = 34 out of 68 cells from 5 mice, *p* < 0.00001 vs. WT, Fisher’s exact test; **Figure 4C**). In contrast, 2 days after the Cbln1 injection, the percentage of PC s showing single EPSCs increased slightly but not significantly (~ 65%, *n* = 46 out of 71 cells from 5 mice, *p* < 0.05 vs. *cbln1*^-/-^ PCs injected with the control solution, Fisher’s exact test; **Figure 4C**). No further significant increase in the percentage of PC s with single EPSCs was seen 4 d after the Cbln1 injection (~ 60%, *n* = 24 out of 40 cells from 3 mice, *p* > 0.1 vs. *cbln1*^-/-^ PCs at 2 days after Cbln1 injection, *n* = 40 cells from 3 mice, Fisher’s exact test; **Figure 4C**). Thus, the CF innervation pattern was only partially rescued by a single Cbln1 injection. Similarly, although transient GluD2 expression restored PF–PC synapses in GluD2^-/-^ mice, it failed to correct the sustained innervation of PC s by multiple CFs (Kohda et al., 2007; Kakegawa et al., 2009), suggesting that rescuing the mature CF innervation pattern may require more time than rescuing PF synapses in both *cbln1*^-/-^ and GluD2^-/-^ mice. These results indicate that acquisition of delay EBC may not require mature CF innervation patterns in the cerebellum.

### PF-LTD, BUT NOT PF-LTP, IS IMPAIRED IN *CBLN1*^-/-^ MICE AND RESCUED BY CBLN1 INJECTION

**FIGURE 5:**
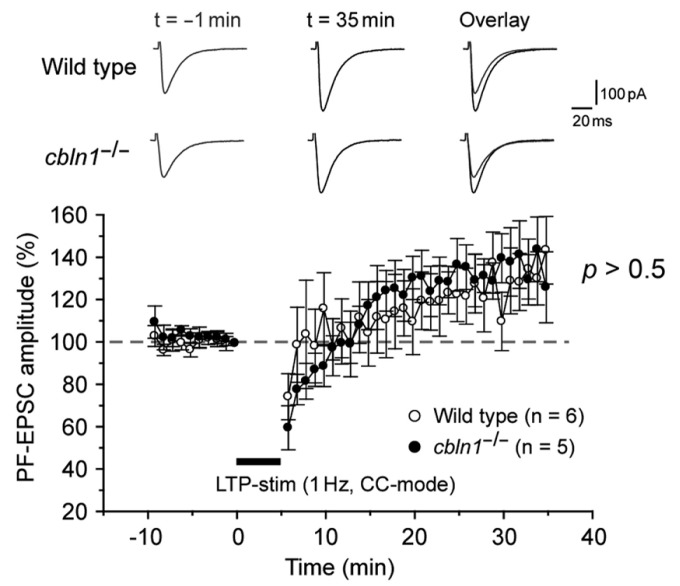
**LTP at PF–Purkinje cell synapses is normally induced in *cbln1*^-/-^mice.** A summary of LTP sessions in cerebellar slices prepared from wild-type and *cbln1*^-/-^mice. The averaged amplitudes of the PF-EPSC over 1 min were normalized to the baseline value, which was the average of the 1 min responses (six traces) that occurred just before an LTP-inducing stimulation (300 times of single PF stimuli at a frequency of 1 Hz in the current-clamp mode). Inset traces show the PF-EPSCs just before (*t* = -1 min) or 35 min (*t* = 35 min) after the LTP stimulation, and their superimposition (overlay). No differences were observed in PF-EPSC amplitudes after the LTP stimulation between wild-type and *cbln1*^-/-^ mice (*p* > 0.5, wild type vs. *cbln1*^-/-^ mice, Mann–Whitney’s *U* test, *n* = 6 for wild type, *n* = 5 for *cbln1*^-/-^ mice).****

Recently, PF-LTP was proposed as a substrate for motor learning in the cerebellum (Schonewille et al., 2010). Thus, we next examined whether PF-LTP could be induced in *cbln1*^-/-^ PC s in acute slice preparations. As reported previously (Lev-Ram et al., 2002; Kakegawa and Yuzaki, 2005), 300 single stimulations of the PF at a frequency of 1 Hz in the current-clamp mode induced postsynaptic PF-LTP in WT PCs (**Figure 5**). Similarly, PF-LTP was normally induced in *cbln1*^-/-^ mice.

**FIGURE 6:**
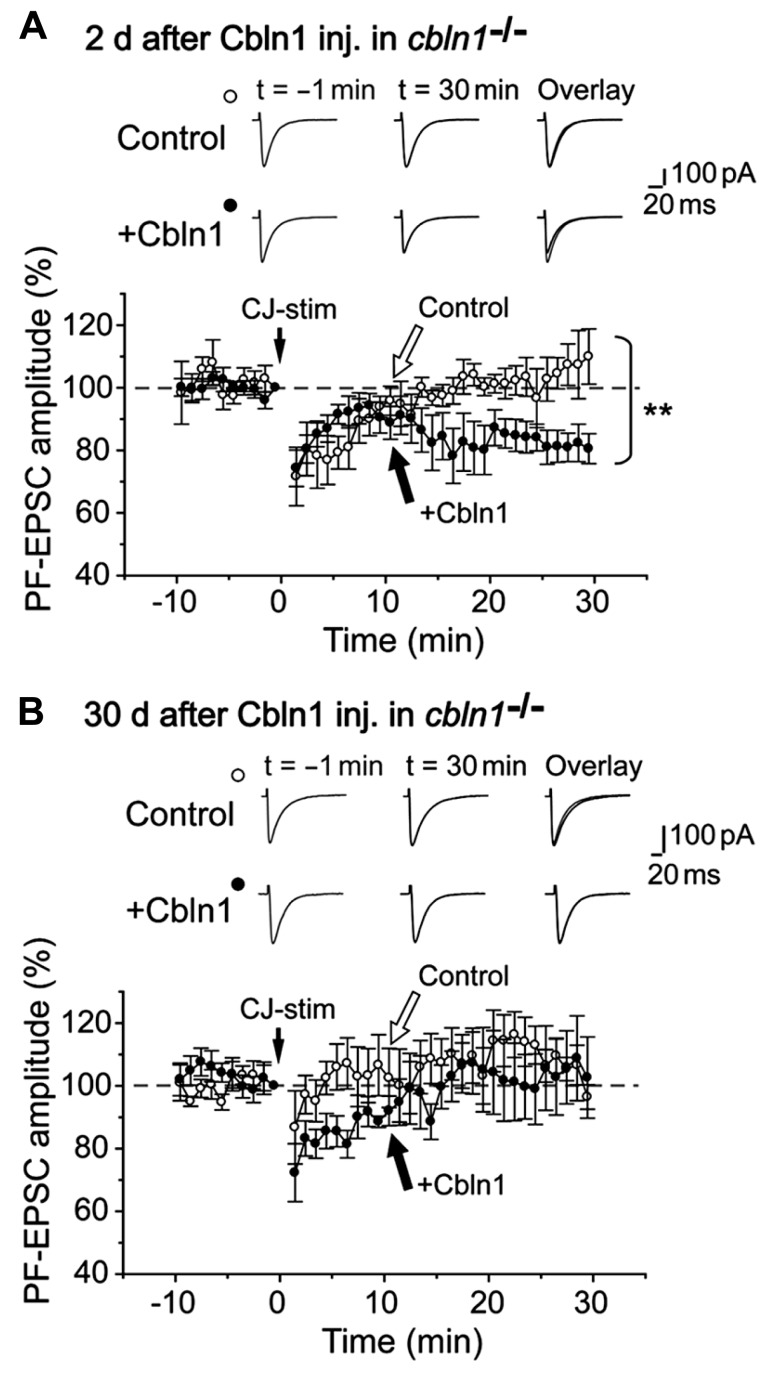
**Subarachnoidal Cbln1 injection transiently restores LTD at *cbln1*^-/-^mouse PF–Purkinje cell synapses.** A summary of LTD sessions in cerebellar slices prepared from *cbln1*^-/-^ mice at 2 days **(A)** and 30 days **(B)** after injection of Cbln1 (+Cbln1), or control solution that did not contain Cbln1 (Control). The averaged amplitudes of the PF–EPSC over 1 min were normalized to the baseline value, which was the average of the 1 min responses (six traces) that occurred just before a conjunctive stimulation (CJ-stim) composed of 30 single PF stimuli together with 200-ms depolarizing pulses from a holding potential of –60 to +20 mV. Inset traces show the PF–EPSCs just before (*t* = –1 min) or 30 min (*t* = 30 min) after the CJ-stim, and their superimposition (overlay). Note that LTD was significantly restored in *cbln1*^-/-^ mice at 2 days after Cbln1 injection (**p < 0.05, control vs. Cbln1, Mann-Whitney’s *U* test, *n* = 5 for control, *n* = 6 for Cbln1), but not at 30 d after injection (*p* > 0.5, control vs. Cbln1, Mann-Whitney’s *U* test, *n* = 5 for control, *n* = 6 for Cbln1).****

In contrast, LTD is impaired at PF–PC synapses in *cbln1*^-/-^ mice (Hirai et al., 2005). To clarify whether this impairment can be rescued by injecting Cbln1, we next prepared cerebellar slices from *cbln1*^-/-^ mice injected with Cbln1. As reported previously, an injection of recombinant Cbln1 rapidly restored PF–PC synaptic transmission (Ito-Ishida et al., 2008). Furthermore, a conjunctive stimulation, which consisted of 30 single stimulations of the PF together with a 200-ms depolarization of the PCs, successfully induced PF-LTD in the *cbln1*^-/-^ PC s 2 days after the Cbln1 injection treatment, but failed to induce PF-LTD in *cbln1*^-/-^ PCs after the injection of the control solution (**Figure 6A**). The PF-EPSC amplitude 25- to 30-min after the combined stimulation was 81 ± 5% (*n* = 6 from five mice) of the control responses in PCs 2 days after a Cbln1 injection, whereas that of cells from mice injected with the control solution was 106 ± 7% (*p* < 0.05, Mann–Whitney’s *U* test, *n* = 5 from 5 mice). In contrast, the conjunctive stimulation failed to induce PF-LTD either in control *cbln1*^-/-^ PCs (injected with the control solution) or in *cbln1*^-/-^ PCs 30 d after Cbln1 treatment (**Figure 6B**). The amplitude of PF-EPSCs 25- to 30 min after stimulation was 105 ± 12% (*n* = 6 from 6 mice) of the control responses in PC s at 30 days after the Cbln1 injection, whereas that of cells treated with the control solution was 105 ± 4% (*p* > 0.5, Mann–Whitney’s *U* test, *n* = 5 from 5 mice). These findings indicate that Cbln1 injection rapidly but transiently restored PF-LTD in *cbln1*^-/-^cerebellum. Furthermore, PF-LTD, but not PF-LTP, was likely to be directly involved in the acquisition of delay EBC in *cbln1*^-/-^ mice.

### CONDITIONED EYEBLINK RESPONSES ARE MAINTAINED AND EXPRESSED IN *CBLN1*^-/-^ MICE

**FIGURE 7:**
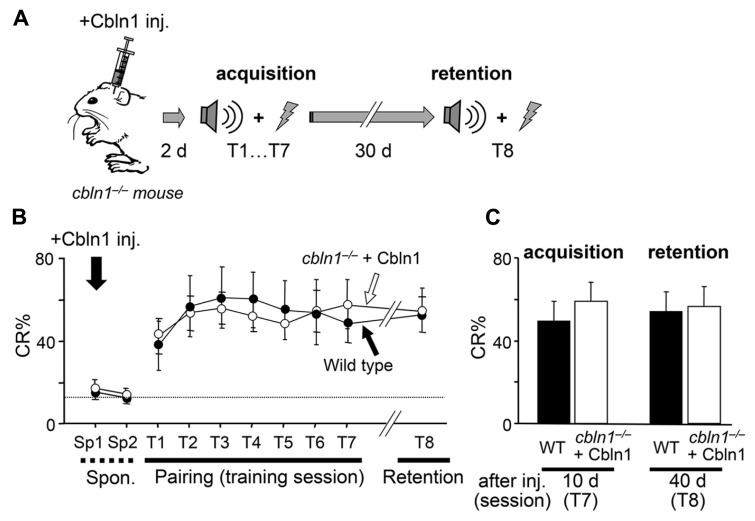
**Intact retention and expression of EBC in *cbln1*^-/-^ mice.** **(A)** A diagram showing a sequence of experiments: *cbln1*^-/-^ mice were injected with Cbln1 as described in **Figure 3** and subjected to delay eyeblink conditioning (EBC) from 2 (T1–T7) and at 40 days (T8, 30-days interval between T7 and T8) after the injection to examine the retention of learned responses. **(B)** Intact retention and expression of EBC in *cbln1*^-/-^ mice. During training (T1–T7) and retention (T8) sessions, no difference in CR% was observed between wild-type mice and *cbln1*^-/-^ mice injected with Cbln1 (*p* > 0.1, a two-way repeated measures ANOVA, *n* = 8 for each group). The dotted line corresponds to the percentage of CR-like activities on Sp2 to indicate the baseline for learned responses. **(C)** The level of CR% was similar between wild-type and *cbln1*^-/-^ mice injected with Cbln1 at T8 as well as at T7 (*p* > 0.1, by Mann-Whitney’s *U* test, *n* = 8 for each group).

The precise role of PF–PC synapses in delay EBC has been unclear (Christian and Thompson, 2003; Boyden et al., 2004). Taking advantage of Cbln1’s ability to rapidly and transiently restore morphological (**Figure 4**) and functional (**Figure 6**; Ito-Ishida et al., 2008) PF synapses, we next examined the roles of PF synapses at various stages of delay EBC. First, we injected Cbln1 into *cbln1*^-/-^ mice and performed delay EBC training 2 days later. After these mice acquired motor learning, as judged by a CR% greater than 60% in the seventh training session (CR% at the seventh session, 63.1 ± 0.1% for wild type vs. 64.2 ± 0.2% for injected *cbln1*^-/-^ mice, *p* > 0.1, Mann–Whitney’s *U* test, *n* = 8 for each group; **Figures 7A, B**), they were kept in their home cages without any further training until the eighth session, 30 days later. Interestingly, *cbln1*^-/-^ mice that received training 2 days after the Cbln1 injection showed high CR% levels at the eighth session, on day 40; these levels were comparable to those of WT mice in the same session (CR% at the eighth session, 58.1 ± 0.2% for WT vs. 60.2 ± 0.1% for injected *cbln1*^-/-^ mice, *p* > 0.1, Mann–Whitney’s *U* test, *n* = 8 for each group; **Figures 7B, C**). In addition, the CR% was similar between the seventh and eighth sessions (*p* > 0.1, Mann–Whitney’s *U* test) in both WT and in *cbln1*^-/-^ mice treated with Cbln1 (**Figures 7B, C**), indicating that Cbln1 was unnecessary for the maintenance and expression of memory traces. However, as shown above, 30 days after the Cbln1 injection, PF–PC synapses had returned to their morphologically (**Figure 4**) and functionally (**Figure 6**) impaired state in *cbln1*^-/-^ mice. In addition, *cbln1*^-/-^ mice that were given a Cbln1 injection but did not receive any training for 30 days failed to acquire associative learning (**Figure 3C**). Together, these results suggest that although intact PF synapses are necessary for acquiring delay EBC, they may be dispensable for its expression and maintenance of previously acquired memory.

### EXTINCTION OF CONDITIONED EYEBLINK RESPONSES DOES NOT REQUIRE CBLN1

**FIGURE 8:**
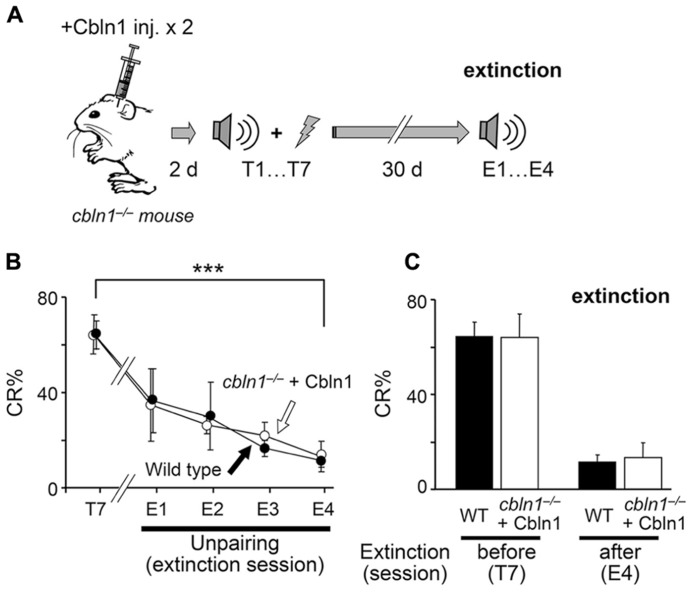
**Conditioned eyeblink responses (CRs) are extinguished in *cbln1*^-/-^ mice.** **(A)** A diagram showing a sequence of experiments: *cbln1*^-/-^ mice were injected with Cbln1 as described in **Figure 3** and trained for delay EBC (T1–T7) beginning 2 days after the Cbln1 injection. From day 40 (30 days after T7), mice were given extinction sessions (E1–E4), in which only a CS tone was given, 100 times per day, to examine the extinction of learned responses. **(B)** Intact extinction of conditioned eyeblink responses in *cbln1*^-/-^ mice. The CR% dramatically decreased in both wild-type mice (*n* = 7) and *cbln1*^-/-^ mice injected with Cbln1 (*n* = 7; ***p < 0.01, T7 vs. E4 in each group, by Mann–Whitney’s *U* test). During extinction sessions (E1–E4), no difference in CR% was observed between wild-type mice and *cbln1*^-/-^ mice injected with Cbln1 (*p* > 0.1, a two-way repeated measures ANOVA, *n* = 7 for each group). **(C)** The CR% was similar between wild-type mice and *cbln1*^-/-^ mice injected with Cbln1 at E4 as well as at T7 (*p* > 0.1, by Mann–Whitney’s *U* test, *n* = 8 for each group).

The memory trace in EBC is known to be rapidly extinguished by repeated exposure to CS-only presentations, but the underlying mechanisms are not completely clear. Thus, using the Cbln1 transient rescue method, we examined PF–PC synapse roles during extinction sessions (**Figure 8A**). We first injected Cbln1 into *cbln1*^-/-^ mice, and performed delay EBC training 2 d later. After these mice acquired motor learning, as judged by a CR% larger than 60% in the seventh training session (CR% at the seventh session, 61.1 ± 0.2% for wild type vs. 62.1 ± 0.1% for injected *cbln1*^-/-^ mice, *p* > 0.5, Mann–Whitney’s *U* test, *n* = 7 for each group; **Figures 8A, B**), they were returned to their home cages and did not receive further training for 30 d, after which they were subjected to 4-day extinction sessions in which only a CS tone was given to the animals 100 times per day. The CR% of both WT and *cbln1*^-/-^ mice was gradually reduced to levels comparable to those prior to training (CR% at the fourth extinction session, 14.0 ± 0.2% for WT vs. to 13 ± 0.1% for injected *cbln1*^-/-^ mice, *p* > 0.5, Mann–Whitney’s *U* test, *n* = 7 for each group; **Figures 8B, C**). These results indicate that the presence of Cbln1 and intact PF–PC synapses may both be dispensable for the extinction of memory traces acquired by delay EBC.

## DISCUSSION

Cbln1 is a recently identified synaptic organizer regulating the morphological and functional integrity of PF–PC synapses in the cerebellum (Yuzaki, 2011). Although *cbln1*^-/-^ mice show motor dyscoordination and an ataxic gait, Cbln1’s role, if any, in learning tasks *in vivo* has not been studied previously. Here, we showed that the delay EBC paradigm, a form of associative motor learning, was impaired in *cbln1*^-/-^ mice (**Figure 1**). Importantly, a single recombinant Cbln1 injection into the subarachnoid space over the *cbln1*^-/-^ cerebellum rapidly restored delay EBC acquisition (**Figure 3**). Immunohistochemical analysis of the whole brain revealed that the injected Cbln1 was restricted to the cerebellum (Ito-Ishida et al., 2008) and its molecular layer (**Figure 3C**). These findings indicate that, although Cbln1 is expressed in extracerebellar structures, such as the olfactory bulb, entorhinal cortex, and thalamus (Miura et al., 2006), the Cbln1 expressed in the cerebellum probably plays an essential role in acquisition of associative motor memory, as measured by the delay EBC protocol.

Recent studies based on genetically modified mice has challenged a widely held assumptions that PF-LTD is the main mechanism underlying motor learning in the cerebellum, such as EBC (Schonewille et al., 2010, 2011). As an alternative hypothesis, PF-LTP was proposed as a substrate for motor learning (Porrill and Dean, 2008; Schonewille et al., 2010). However, we showed that PF-LTP was normally induced (**Figure 5**), while PF-LTD was impaired in *cbln1*^-/-^ mice (**Figure 6**). Furthermore, impaired PF-LTD was rescued by injection of recombinant Cbln1 (**Figure 6**). These results indicate that although it remains unclear whether and how PF-LTD serves as a memory trace (Hesslow et al., 2013), PF-LTP is dispensable for acquisition of delay EBC at least in *cbln1*^-/-^ mice.

### CBLN1 AS A TOOL TO STUDY PF–PURKINJE CELL SYNAPSE ROLES IN MOTOR LEARNING

The mechanisms underlying delay EBC has been characterized has been extensively studied in rabbits as well as cats, ferrets and rats by lesioning or pharmacological methods (Hesslow and Yeo, 2002; Christian and Thompson, 2003). Nevertheless, the exact role played by the various cerebellar synapses at different learning phases has not been completely clear, partly due to the difficulty of manipulating specific synapses by lesioning or pharmacological methods (Christian and Thompson, 2003; Boyden et al., 2004). Although gene targeting in mice has clarified the involvement of specific genes in delay EBC (Aiba et al., 1994; Kishimoto et al., 2001a; Miyata et al., 2001; Koekkoek et al., 2003, 2005), the location of the synapses responsible for storing the associative memory has remained unclear, because the same gene product is often used by different synapses in the targeted neuron. As discussed in the Section “Introduction,” many molecules, such as PKC, metabotropic glutamate receptor 1, and calcineurin, are not specifically expressed at PF–PC synapses (Yuzaki, 2012). Thus, temporally and spatially controlled gene ablation or expression is particularly important to prevent compensation by backup pathways. Reversible neurotransmission blocking (RNB) techniques using transiently expressed tetanus toxin light chain in cerebellar granule cells can precisely target specific presynaptic terminals in the cerebellar circuits (Wada et al., 2007). However, like pharmacological AMPA receptor blockers, RNB blocks excitatory neurotransmission at PF–interneuron synapses as well as at PF–PC synapses. In addition, since the effect of RNB was assessed by spontaneous simple spikes under general anesthesia, it is unclear whether or to what extent synaptic transmission is inhibited in PC s during EBC. In contrast, we showed here that injected Cbln1 likely regulates synaptic transmission and plasticity specifically at PF–PC synapses. Although Cbln1 is also expressed in the DCN, recombinant Cbln1 injected into the subarachnoid space does not reach the DCN in *cbln1*^-/-^ mice (**Figure 3C**; Ito-Ishida et al., 2008). In addition, GluD2, a postsynaptic receptor for Cbln1 (Matsuda et al., 2010), is not expressed in PC axons (Matsuda et al., 2008) or by other neurons innervating the DCN. Furthermore, although GluD2 is expressed at very low levels in cerebellar interneurons, synaptic transmission at the PF–interneuron synapses is not affected in *GluD2*^-/-^ mice (Yamasaki et al., 2011). Therefore, although EBC in mice has certain limitations (Boele et al., 2010), a rescue approach using a single injection of recombinant Cbln1 serves as a unique and powerful tool to study the specific roles of PF–PC synapses at different learning phases *in vivo*.

### CBLN1 IS REQUIRED FOR THE ACQUISITION, BUT NOT MAINTENANCE OR EXTINCTION OF DELAY EBC

A single injection of recombinant Cbln1 into the *cbln1*^-/-^ cerebellum transiently restored the ability to acquire delay EBC (**Figure 3**). Similarly, a single injection of Cbln1 only transiently morphologically (**Figure 4**) and functionally (**Figure 5**) restored normal PF–PC synapses in *cbln1*^-/-^ mice. These results are consistent with earlier lesion and inactivation experiments targeting the cerebellar cortex in rabbits (Attwell et al., 2002; Christian and Thompson, 2003), suggesting that cerebellar cortical circuits are crucial for acquiring certain CRs. Interestingly, the impaired delay EBC was rescued in *cbln1*^-/-^ mice even when PC s were innervated by multiple CFs (**Figure 4C**). Similarly, we previously showed that ataxic gait and motor dyscoordination (as measured using the rotor-rod test) were significantly rescued by transiently expressed GluD2 in GluD2^-/-^ mice, which maintained multiple-CF innervations of PC s (Kakegawa et al., 2009). Together, these findings indicate that the CF innervation pattern may not play a major role, but intact PF–PC synapses are indispensable for acquisition of memory in the delay EBC paradigm.

Interestingly, we found that *cbln1*^-/-^ mice that received training that began 2 days after a Cbln1 injection retained normal delay EBC responses, even when their PF–PC synapses became morphologically and functionally impaired by Cbln1 loss (**Figure 7**). This finding could be explained by the two-region memory hypothesis (Thompson and Krupa, 1994; Medina et al., 2001): a memory trace is initially formed at PF–PC synapses in *cbln1*^-/-^ mice when training starts, 2 days after the Cbln1 injection. The memory trace is then transferred to the DCN or other regions over time. Similarly, inactivating the cerebellar cortex by a cooling probe impairs CR acquisition, but does not disrupt CR expression in well-trained animals (Clark et al., 1997). Although plastic changes at DCN synapses reportedly encode trained response amplitudes (Garcia and Mauk, 1998; Koekkoek et al., 2003), it is difficult to evaluate this aspect in the present study using EMG recordings.

Alternatively, since approximately 20% of PF–PC synapses were morphologically normal in *cbln1*^-/-^ mice at 30 d after the Cbln1 injection (**Figure 4**), this finding could be explained by the one-region cortical memory hypothesis (Attwell et al., 2002; Kalmbach et al., 2010) in which a memory trace, probably formed as LTD at the PF–PC synapses in *cbln1*^-/-^ mice, when training begins 2 days after the Cbln1 injection, and 30 days after the injection the remaining PF synapses may retain enough information to express CRs. In either case, our findings indicate that the presence of Cbln1 and a large majority of the PF–PC synapses (and CF synapses) are unnecessary for maintaining and expressing delay EBC memory traces.

The normal extinction of the EBC that was acquired by *cbln1*^-/-^ mice after the Cbln1 injection, even though they had few normal PF–PC synapses remaining (**Figure 8**), is consistent with the one-region cortical memory hypothesis, because inhibition of CF activities is shown to serve as a teaching signal for extinction by reversing LTD in the cerebellar cortex (Medina et al., 2002). In this scenario, the memory trace was formed at PF– PC synapses in *cbln1*^-/-^ mice, as training began 2 days after the Cbln1 injection, maintained in the remaining synapses at 30 days, and removed by reduced CF activity during extinction training. However, the alternative hypothesis that the memory trace is actually stored in regions outside of the cerebellar cortex and is removed during extinction training cannot be ruled out. Although further studies are required to clarify the extinction mechanisms, it is clear that the presence of Cbln1 and intact PF–PC synapses are not likely to be necessary for it.

Inactivating specific synapses could cause imbalances in the overall performance of cerebellar networks, leading to non-specific impairment in motor learning (Bracha et al., 2009). However, our findings that the PF–PC synapses played different roles in acquiring, maintaining, and expressing delay EBC indicate that PF–PC synapses are indeed involved in specific aspects of EBC. This study also establishes the use of a single injection of recombinant Cbln1 as a tool for studying the roles of PF–PC synapses in different stages and paradigms of motor learning. Finally, since LTD and delay EBC become impaired in aged mice (Woodruff-Pak et al., 2010), and these functions were improved in this study by injecting recombinant Cbln1 into the cerebellar subarachnoid space, this molecule may have potential as a therapeutic agent for improving motor performance in elderly people.

## AUTHORS CONTRIBUTION

Kyoichi Emi, Wataru Kakegawa, Kazuhisa Kohda, and Michisuke Yuzaki designed the experiments. Kyoichi Emi and Kazuhisa Kohda performed behavioral studies. Wataru Kakegawa performed electrophysiological experiments. Aya Ito-Ishida prepared recombinant HA-Cbln1. Eri Miura performed electron microscopic analyses. Kyoichi Emi, Wataru Kakegawa, Kazuhisa Kohda, and Michisuke Yuzaki wrote the paper.

## Conflict of Interest Statement

The authors declare that the research was conducted in the absence of any commercial or financial relationships that could be construed as a potential conflict of interest.
